# Is a history of episodic low back pain an indicator of Modic changes?

**DOI:** 10.1016/j.inpm.2023.100239

**Published:** 2023-03-16

**Authors:** Josh Levin, Derek Schirmer, Roxana Garcia, David Levi

**Affiliations:** a Department of Orthopaedic Surgery, Stanford University, 450 Broadway St., Pavilion C, 4^th^ Floor, MC 6342, Redwood City, CA, 94063, USA; b Department of Neurosurgery, Stanford University, USA; c Jordan Young Institute, Virginia Beach, VA, 5716 Cleveland St., Suite 200, Virginia Beach, VA, 23462, USA

## Abstract

**Background:**

Prior work demonstrated that a history of episodic low back pain was highly indicative of discogenic pain. Recently, there has been more focus on vertebrogenic pain, however little is known about the clinical features of this condition.

**Purpose:**

To determine if a history of severe episodic low back pain correlates with Modic endplate changes on lumbar spine magnetic resonance imaging (MRI), presumed to be a marker of vertebrogenic pain.

**Study design:**

/setting: Retrospective, observational, in vivo study of consecutive patients at outpatient Physical Medicine & Rehabilitation clinics at a single academic spine center.

**Patient sample:**

Consecutive patients who received a lumbar spine MRI between January 1, 2020, and December 31, 2020.

**Methods:**

A retrospective chart review identified patients who received a lumbar spine MRI in 2020. Chart review then determined if patients had a history of episodes of low back pain lasting at least 2 days, or if they had non-episodic low back pain (pain beginning with a gradual onset or after a specific event with continuous symptoms for >3 months). Patients were excluded if they had prior lumbar spine surgery, radicular leg pain without low back pain, indeterminate presentations based on chart review, acute spine fractures, or metastatic spine lesions. For the primary analysis, the MRIs were reviewed and were dichotomized into positive (having for either type 1 or type 2 Modic changes at any level) or negative (no Modic changes at any level).

**Results:**

A total of 111 patients were analyzed. Inter-rater reliability for determining whether a patient's low back pain was episodic was strong (kappa ​= ​0.83), as was inter-rater reliability for determining if a patient had any levels with type 1 or type 2 Modic changes (kappa ​= ​0.81). Seventy-one out of 111 patients had type 1 and/or type 2 Modic changes at one or more spinal levels. The sensitivity of the test (episodic vs non-episodic low back pain) in finding patients with Modic changes was 20% and the specificity was 70%. The diagnostic confidence odds were 1.2, with a diagnostic confidence of 55%. Subgroup analyses for type 1 Modic changes, and for type 2 Modic changes, showed similar values.

**Conclusions:**

A history of episodic low back pain is not a strong indicator for a vertebrogenic etiology.

## 1 Introduction

Chronic low back pain (LBP) is highly prevalent and associated with a significant burden of care and cost nationally and globally [1,2]. Despite the disabling impact that LBP has on society, in some clinical practices, 80–90% of LBP cases are identified as “non-specific” [2]. Improved differentiation of the etiology of LBP is important to identify patients who may be appropriate candidates for treatment. Largely due to the diagnostic challenges, the effect size of treatments for LBP is only small to moderate, highlighting the need for better diagnostic algorithms which may lead to more effective treatments [3].

Historically, the intervertebral disc has been shown to be responsible for approximately 40% of chronic low back pain [4]. Disc disruption is associated with annular fissures and abnormal ingrowth of sinuvertebral nerve fibers into these fissures, leading to nociception [[5], [6], [7]]. However, recent studies have shown that fissures and nerve ingrowth into discs may be less common than vertebral body endplate defects in patients with chronic LBP [8]. The concept of vertebrogenic pain led to a new structural model of low back pain focused on the vertebral body endplates [9] which are innervated by the basivertebral nerve [8,10,11]. Endplate changes are more easily identified on histology [8], but are also visualized as Modic changes on MRI [12], and these imaging findings are highly correlated with low back pain [13,14]. Growing evidence suggests that patients with Modic changes may benefit from basivertebral nerve ablation [9,15,16].

Although type 1 and type 2 Modic changes may be associated with increased severity and disability of LBP, there are few studies that have elucidated the history of LBP further [14,[17], [18], [19], [20]]. Episodic low back pain was previously shown to be indicative of a discogenic etiology based upon correlation with positive discography [21]. Since the intervertebral disc and the vertebral body endplates are in close proximity to one another in the anterior aspect of the spine, we sought to determine if a history of severe episodic low back pain was also indicative of a vertebrogenic etiology as measured by the presence of type 1 or type 2 Modic changes on MRI.

## 2 Methods

This study was approved by our academic medical center's institutional review board (IRB #58932) as a portion of a larger single site retrospective observational study. The study was conducted according to the Declaration of Helsinki. A database search identified all patients who had presented to one spine physiatrist's outpatient clinic and had received a lumbar spine MRI between January 1, 2020, and December 31, 2020. A chart review was then performed to determine the presence or absence of Modic changes and to identify whether the patients' low back pain was episodic or not.

It is recognized that currently there is no gold standard to diagnose vertebrogenic LBP. Basivertebral nerve ablation has been used to treat LBP of this presumed source based upon the criteria of type 1 or type 2 Modic changes [9,15,16]. In light of this, we used type 1 and type 2 Modic changes as a proxy for a gold standard diagnosis for vertebrogenic LBP.

Inclusion criteria were all patients aged ≥18 who underwent a lumbar spine MRI during the year 2020, with images available in our electronic medical record system. Exclusion criteria were patients with radicular leg pain without low back pain, patients with acute low back pain without a previous history of low back pain, prior lumbar spine surgery, acute spine fractures, and metastatic spine lesions.

## 3 MRI review

All lumbar spine MRIs were reviewed independently by two investigators (JL and DS). Each investigator evaluated all spinal levels between L1-2 and L5-S1 for the presence or absence of type 1 or type 2 Modic changes. For the primary analysis, patients were dichotomized into positive (either type 1 or type 2 Modic changes at any level) or negative (no Modic changes at any level). Secondary analyses were performed for type 1 Modic changes and for type 2 Modic changes. Inter-rater reliability was calculated, and a consensus evaluation was performed for all disagreements.

## 4 Chart review

We used the same definition of episodic low back pain that was used in our prior work [21]. In short, episodic pain was defined as pain that began with a severe episode, followed by intermittent disabling episodes lasting longer than two days. Non-episodic low back pain was again defined as pain that began with a gradual onset, or pain that developed after a specific event with continued symptoms [21]. Each chart was reviewed independently by two investigators (DS and RG), and inter-rater reliability was calculated. A third investigator (JL) independently served as a tiebreaker for disagreements.

## 5 Statistical analysis

Inter-rater reliability was assessed by calculating the simple kappa statistic with a 95% confidence interval (CI). Agreement was defined as almost perfect for kappa above 0.90, strong for 0.80 to 0.90, moderate for 0.60 to 0.79, weak for 0.40 to 0.59, minimal for 0.21 to 0.39, and none for 0 to 0.20 [22].

For the primary analysis, patients were dichotomized into positive for Modic changes (type 1 and/or type 2 Modic changes at any level between L1-2 and L5-S1) or negative for Modic changes (no Modic changes at any level). Secondary analyses were performed in which patients were dichotomized into positive for type 1 Modic changes (type 1 Modic changes at any level between L1-2 and L5-S1) or negative for type 1 Modic changes (no type 1 Modic changes at any level). If a patient had type 1 Modic changes, and also had type 2 Modic changes (either mixed type 1/type 2 Modic changes at the same level, or type 2 Modic changes at a different level), they were considered positive for type 1 Modic changes. A similar analysis was performed for type 2 Modic changes. For each analysis, sensitivity, specificity, positive likelihood ratio, and prevalence odds were calculated in order to calculate the diagnostic confidence odds.

## 6 Results

152 patients underwent a lumbar spine MRI during the 1-year time period. In total, 41 patients were excluded for the following reasons: prior lumbar spine surgery (18), radicular leg pain without low back pain (9), indeterminate clinical presentation (7), acute spine fracture (5), and metastatic spine lesion (2). 111 patients remained and were included in the final analysis. See Table 1 for demographic patient information.

**Table 1:** Baseline demographic information, presented as n (%) for categorical variables, and means and standard deviations for continuous variables.

Female	56 (50%)
Age (years)	59 (SD ​= ​17)
BMI	27 (SD ​= ​7)

Inter-rater reliability for determining whether a patient's pain was episodic or not was strong with a kappa value of 0.83 [95%CI: 0.70–0.96]. Inter-rater reliability for the primary analysis of determining if a patient had any Modic changes at any level (n ​= ​111) was also strong with a kappa value of 0.81 [95%CI: 0.70–0.92]. Inter-rater reliability for determining the agreement of each disc separately (n ​= ​555) was moderate with a kappa value of 0.70 [95%CI: 0.62–0.78].

For the primary analysis, 71 patients (64%) were positive for type 1 and/or type 2 Modic changes at any level, while 40 patients were negative. Using Modic changes as the gold standard diagnosis (which was presumed to represent vertebrogenic pain), and the history of episodic low back pain as the diagnostic test, the sensitivity was 20%, the specificity was 70%, and the positive likelihood ratio was 0.67. The positive predictive value (PPV), based upon a prevalence of 64%, was 54%. The diagnostic confidence odds were 1.2, with a diagnostic confidence of 55%. See Table 2.

**Table 2:** Patients who were positive for type 1 and/or type 2 Modic changes at any level.

Prevalence	71/111 (64%)
Sensitivity	20%
Specificity	70%
Positive likelihood ratio	0.67
Positive predictive value	54%
Diagnostic confidence odds	1.2
Diagnostic confidence	55%

For the secondary analysis evaluating type 1 Modic changes, 34 patients (31%) were positive while 77 were negative. The sensitivity was 24%, the specificity was 78%, and the positive likelihood ratio was 1.06. The PPV, based upon a prevalence of 31%, was 32%. The diagnostic confidence odds were 0.5, with a diagnostic confidence of 32%. See Table 3.

**Table 3:** Patients who were positive for type 1 Modic changes at any level.

Prevalence	34/111 (31%)
Sensitivity	24%
Specificity	78%
Positive likelihood ratio	1.06
Positive predictive value	32%
Diagnostic confidence odds	0.5
Diagnostic confidence	32%

For the secondary analysis evaluating type 2 Modic changes, 55 patients (50%) were positive while 56 were negative. The sensitivity was 18%, the specificity was 71%, and the positive likelihood ratio was 0.63. The PPV, based upon a prevalence of 50%, was 38%. The diagnostic confidence odds were 0.63, with a diagnostic confidence of 39%. See Table 4.

**Table 4:** Patients who were positive for type 2 Modic changes at any level.

Prevalence	55/111 (50%)
Sensitivity	18%
Specificity	71%
Positive likelihood ratio	0.63
Positive predictive value	38%
Diagnostic confidence odds	0.63
Diagnostic confidence	39%

## 7 Discussion

As the understanding of vertebrogenic low back pain has advanced, a paradigm shift has taken place in both the diagnostic and therapeutic approaches to low back pain. Historically, the treatment options for patients with axial low back pain due to anterior element pathology have been limited. However, for the subset of patients with type 1 and/or type 2 Modic changes, and a presumed vertebrogenic etiology of their symptoms, basivertebral nerve ablation has been shown to be an effective treatment [9,15,16].

For any treatment to be successful, appropriate patient selection is necessary. For basivertebral nerve ablation, imaging features other than Modic changes have not been shown to significantly affect outcomes [23]. Pain on lumbar extension has been associated with type 1 Modic changes [17], and a midline location of low back pain correlates with better outcomes from basivertebral nerve ablation [20]. Yet many patients with various etiologies of their pain have midline low back pain [24], and Modic changes are present in approximately 6% of asymptomatic individuals [14]. This makes the selection of patients for treatment with basivertebral nerve ablation challenging, especially given its more invasive nature compared to other percutaneous spine procedures. While several studies have shown treatment from basivertebral nerve ablation to be effective, many patients who undergo the procedure do not have success. Given the multitude of failures from spine treatments throughout the years, several physicians have understandably shown excitement for the 66% long-term success rate from this newer treatment (≥50% reduction of pain) [16]. However, some physicians may neglect to clarify with patients that a 66% success rate is equivalent to a 34% failure rate. Patients may not necessarily view this, nor the 34% rate of complete resolution of pain [16], so favorably. Therefore, understanding additional features of vertebrogenic pain may be helpful in future patient selection in order to improve success rates from treatment even further.

Since our prior work demonstrated that a history of episodic axial low back pain was highly indicative of a discogenic etiology [21], and given the proximity of the disc to the vertebral bodies, we presumed that vertebrogenic low back pain may present similarly. If so, this simple historical feature of a patient's clinical presentation might play a role in patient selection for basivertebral nerve ablation. However, the current study demonstrates that this is not the case. Our 55% diagnostic confidence for patients with type 1 and/or type 2 Modic changes at any level demonstrates that a history of episodic low back pain is essentially no better than a coin flip in determining a vertebrogenic etiology of their symptoms. Our subgroup analyses of type 1 and type 2 Modic changes separately showed even lower diagnostic confidence values.

The lack of an association between a history of episodic LBP and a vertebrogenic etiology may have clinical implications. Although midline axial low back pain may be presumed to be an indication of a vertebrogenic source of pain based upon the response to basivertebral nerve ablation [20], there is also evidence that midline LBP is a specific finding for a discogenic source of LBP [24]. Although there appears to be no association between episodic LBP and type 1 and type 2 Modic changes, it is unclear if a history of episodic LBP would serve as a negative prognostic factor for basivertebral nerve ablation in an individual with these endplate changes. It is also unknown if an individual with Modic changes and positive discography would be less likely to have success from basivertebral nerve ablation. These may be areas for future research.

Limitations to our study include the retrospective study design, and the inherent biases in patient selection with retrospective studies. We did include all consecutive patients, which can limit these biases. The strongest limitation, however, is the lack of a true gold standard diagnosis for vertebrogenic low back pain. While type 1 and type 2 Modic changes are highly associated with basivertebral nerve ablation success [9,15,16], Modic changes also are present in asymptomatic individuals as well as other disease processes [13,14]. Therefore, our use of Modic changes as a proxy for vertebrogenic low back pain is a significant limitation. Finally, the sample size of 111 patients was small, and all data was from a single clinical practice. As such, the results may not be generalizable to other clinical settings.

## 8 Conclusions

Unlike discogenic pain, a history of episodic low back pain does not appear to be indicative of a vertebrogenic etiology, and should not be used as a selection criteria for basivertebral nerve ablation.

## Declaration of competing interest

The authors declare the following financial interests/personal relationships which may be considered as potential competing interests:

Josh Levin reports a relationship with Scilex Pharmaceuticals that includes: consulting or advisory.
